# Exploring brain activity for positive and negative emotions by means of EEG microstates

**DOI:** 10.1038/s41598-022-07403-0

**Published:** 2022-03-01

**Authors:** Giulia Prete, Pierpaolo Croce, Filippo Zappasodi, Luca Tommasi, Paolo Capotosto

**Affiliations:** 1 Department of Psychological, Health and Territorial Sciences, “G. d’Annunzio” University of Chieti-Pescara, Blocco A, Via dei Vestini 29, 66100 Chieti, Italy; 2 Department of Neuroscience, Imaging and Clinical Sciences, “G. d’Annunzio” University of Chieti-Pescara, Via dei Vestini 33, 66100 Chieti, Italy; 3 Institute for Advanced Biomedical Technologies, “G. d’Annunzio” University of Chieti-Pescara, Via Luigi Polacchi 11, 66100 Chieti, Italy

**Keywords:** Cognitive neuroscience, Emotion

## Abstract

Microstate analysis applied to electroencephalographic signals (EEG) allows both temporal and spatial imaging exploration and represents the activity across the scalp. Despite its potential usefulness in understanding brain activity during a specific task, it has been mostly exploited at rest. We extracted EEG microstates during the presentation of emotional expressions, presented either unilaterally (a face in one visual hemifield) or bilaterally (two faces, one in each hemifield). Results revealed four specific microstate’s topographies: (i) M1 involves the temporal areas, mainly in the right hemisphere, with a higher occurrence for stimuli presented in the left than in the right visual field; (ii) M2 is localized in the left temporal cortex, with higher occurrence and coverage for unilateral than bilateral presentations; (iii) M3, with a bilateral temporo-parietal localization, shows higher coverage for bilateral than unilateral presentation; (iv) M4, mainly localized in the right fronto-parietal areas and possibly representing the hemispheric specialization for the peculiar stimulus category, shows higher occurrence and coverage for unilateral stimuli presented in the left than in the right visual field. These results suggest that microstate analysis is a valid tool to explore the cerebral response to emotions and can add new insights on the cerebral functioning, with respect to other EEG markers.

## Introduction

Emotions are complex inputs to our brain, and although a very large number of studies have tried to frame the cerebral activity associated with emotional encoding, the resulting scenario is still fragmented, with different findings often in contradiction with each other (for a recent meta-analysis see^1^). An unresolved issue in this domain concerns hemispheric asymmetry in processing different emotions (see^2–5^). As a matter of fact, if some studies described a right-hemispheric superiority for all emotions (Right Hemisphere Hypothesis, RHH^6,7^), other evidence revealed a left- vs right-hemispheric superiority for positive vs negative valence stimuli, respectively (Valence Hypothesis, VH^8,9^). To disentangle this issue, at a behavioural level, methodological expedients have been exploited, such as the divided visual field paradigm, in which stimuli are presented lateralized in one visual field, based on the contralateral projections of the human visual system^10^: a stimulus presented in the Left Visual Field (LVF) reaches primarily the right hemisphere (and vice versa) if it is presented for a time shorter than that needed to act a saccadic movement (about 150 ms). By using this paradigm, it is possible to indirectly investigate cerebral competencies for lateralized presented stimuli. For instance, in a study, emotional faces were briefly presented either in the LVF or in the Right Visual Field (RVF) and observers were asked to rate how friendly each face appeared^11^. By means of this procedure, it was found that emotional faces presented in the RVF (left hemisphere) were judged as more friendly than those presented in the LVF (right hemisphere), thus confirming the valence-specific specialization of the two hemispheres. In a more complex version of the divided visual field paradigm, two stimuli can be presented simultaneously, one in each hemifield: if they convey different emotions, and the observer is asked to express a single emotional judgment, it is possible to infer which hemisphere is dominant in that task (e.g., the right hemisphere, if the emotional judgment is based on the valence of the stimulus shown in the LVF). Using the same friendliness evaluation task as described above, it was found that when two emotional faces expressing two opposite-valence emotions (angry and happy) were presented simultaneously, participants based their responses mainly on the emotional content of the stimulus shown in the LVF, independently of its positive or negative valence, thus supporting a right-hemispheric superiority for all emotions^12^. Again, the pattern of results found by using the unilateral vs bilateral presentation of emotional faces was contradictory enough to prevent conclusively clarifying the role of the two halves of the brain in emotion processing (see also^13,14^).

In this scenario, electroencephalographic (EEG) studies involving emotional stimuli are helpful in elucidating the hemispheric involvement in both the perception of emotion (i.e., perceiving a happy face^15^) and the behavioral components associated to emotions (i.e., kissing a person^16^). Specifically, Event-Related Potentials (ERPs) for emotional stimuli confirm that peculiar components are temporally and topographically associated with the processing of such input. For instance, starting from the well-established frontoparietal network involved in face perception, widely confirmed by using different electrophysiological techniques^17–20^, specific ERPs have been found to be associated to the emotional valence of face stimuli. In particular, a positive peak recorded at about 100 ms from the stimulus onset (P1) has been suggested to be related to the fast processing of emotional valence, and a following positive peak, recorded at about 200 ms (P2), has been related to a higher-order emotion detection. Both ERP components are stronger in the frontoparietal areas of the right than the left hemisphere when emotional stimuli are presented centrally^21^. By using the same divided visual field described above, during EEG recording, we reported that the P1 component showed a higher amplitude in the right than in the left hemisphere during the unilateral presentation of emotional faces, regardless of the positive or negative valence, and that the P2 component showed a higher amplitude in the right hemisphere for both unilateral and bilateral presentations of emotional faces, thus confirming the main involvement of the right hemisphere in all emotion processing^22^. Nevertheless, in the same study we also reported that the emotional judgments expressed by participants in the task were more positive for emotional faces presented in the RVF (left hemisphere) and more negative for emotional faces presented in the LVF (right hemisphere), regardless of their happy or angry expression^22^, thus providing evidence for a valence-specific specialization of the cerebral hemispheres. This contrasting pattern of results between ERPs and behavioural responses highlights once again the complex frame of cerebral processing for positive and negative emotions. Furthermore, connectivity analysis on the same data^15^ revealed a strong pattern of connectivity among different areas, mainly in the right hemisphere, for both unilateral and bilateral conditions, thus suggesting a right-hemispheric superiority. However, a stronger pattern of connectivity was found in particular when positive/negative emotions were directly presented to the left/right hemisphere, respectively, thus suggesting that the presentation of positive/negative emotions to the left/right hemisphere represents a kind of optimal stimulation for the brain^15^. To sum up, even when using the same paradigm, the specific role of each hemisphere in processing positive and negative emotions remains unresolved, with different methodologies and analyses supporting different patterns of results.

A possible solution to this complex state of things is to consider different kinds of analyses of the same EEG signals that do not investigate classical EEG marker in both temporal (i.e., evoked potential) or frequency (i.e., oscillations) domain. In this scenario a promising way to globally represent the temporary brain activity resulting from concomitant active networks is the microstate analysis. Specifically, EEG microstates represent periods of quasi-stability of the scalp topography for brief windows of time (80–120 ms) in which the electric potential configuration on the scalp remains stable whereas the power of the potential may vary^23–25^. In this way, the EEG signal is reduced to a sequence of stable map topographies over time. Such a topographical approach does not require any type of a priori hypothesis, so that it represents an optimal tool to shed light on the issue of cerebral activity for emotional stimuli.

By exploiting this analysis, four typical topographies have been found to explain about 80% of the EEG variance in healthy adults at rest^26^: the so-called A and B topographies are mirror images of each other and extend from occipital-parietal areas of one hemisphere to the frontal areas of the opposite hemisphere; C topography has a prefrontal orientation; D has an occipital-to-frontal direction. These microstates are suggested to be functionally associated with the auditory, visual, saliency, and attention networks described in resting state fMRI studies^27^. Even if microstates analysis has been mainly used at rest, it is possible to apply it also to a specific task, in order to investigate the cerebral responses to a particular condition. For instance, it has been found that ERPs for emotional words are linked to a distinct pattern of microstates when compared to neutral words, even if no difference was found for different valence or arousal levels^28^. Similarly, Gianotti et al.^29^ applied microstates analysis to a paradigm with emotional stimuli, and they found microstate differences from 140 to 330 ms for valence and from 300 to 520 ms for arousal (with respect to the stimulus onset), a result that extended previous knowledge obtained exploiting classical ERP analysis.

Starting from these premises, this study aimed at applying microstate analysis to EEG data recorded during unilateral and bilateral presentation of faces expressing positive and negative emotions. In particular, starting from the paradigm described in Prete et al.^22,30^, we aimed at investigating microstates related to the presentation of one angry or happy faces presented in either the LVF or the RVF, as well as to the presentation of two emotional faces (happy and/or angry) presented one in each visual field. We believe this innovative analysis approach can help to shed more light on the unresolved field of hemispheric processing of positive and negative emotional stimuli. Due to the scarcity of literature in this domain, we did not start with an a priori hypothesis, being confident that a data-driven approach could offer us the microstate cortical topographies associated with emotional processing, showing their distribution, orientation and timing. However, at a more general level, we speculated that if the right hemisphere is superior than the left hemisphere in all emotion processing, as proposed by the Right Hemisphere Hypothesis^6,7,31^, microstates should be longer (higher duration), less frequent (higher occurrence) and more stable (longer coverage) when one emotional face is presented in the LVF than in the RVF, being happy or angry. Alternatively, according to the Valence Hypothesis^8,9^, we would expect longer, less frequent and more stable microstates when a happy face is presented in the RVF (left hemisphere) and/or an angry face is presented in the LVF (right hemisphere). Considering previous ERP evidence using the same paradigm^22^, however, we expected to find a stronger right-hemispheric involvement in emotion processing, in accordance with the RHH.

## Results

### Microstates source analysis

**Figure 1:**
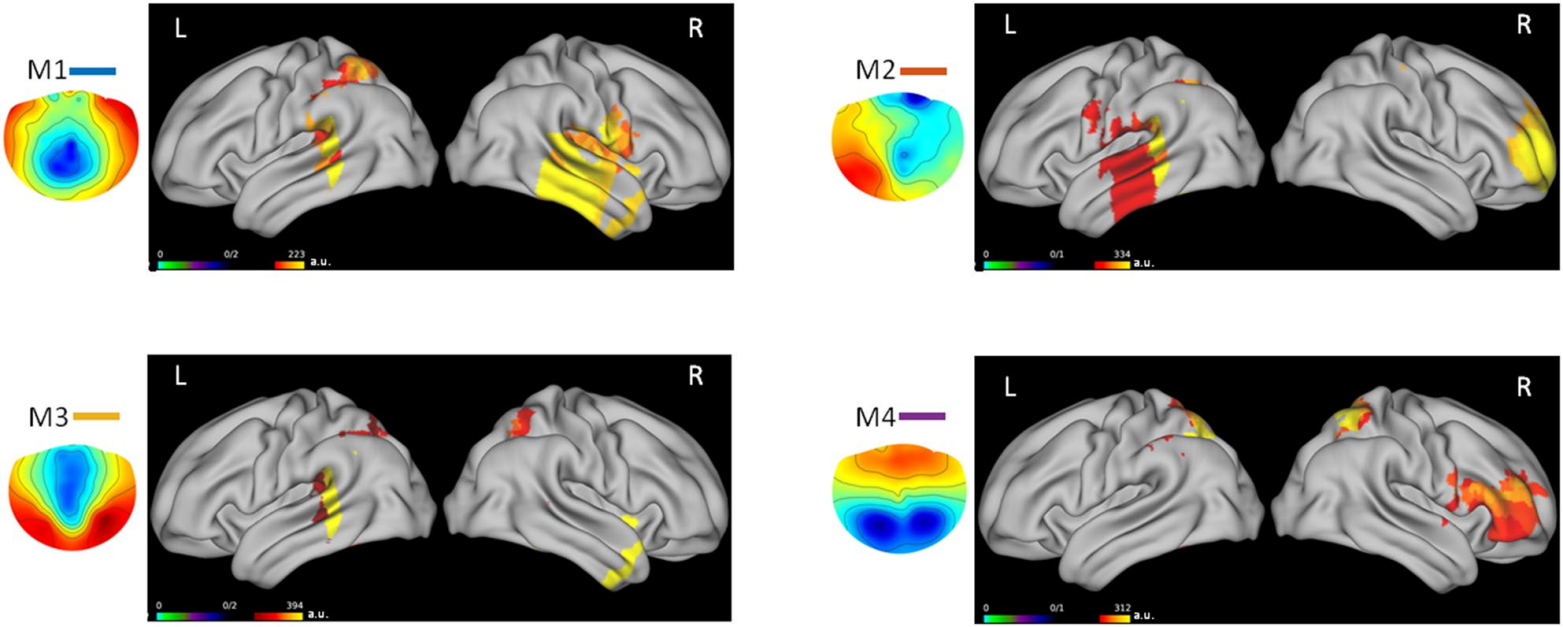
Topography for each microstate (M1, M2, M3, M4). Colors represent left (L) and right (R) hemispheric activity, in arbitrary unit (a.u.).

Figure 1 shows the localized source activity for each microstate’s topography (M1, M2, M3, M4). In particular it can be observed that: (i) M1 involves the temporal areas and it appears to be mainly localized in the right hemisphere, involving the Fusiform Face Area, specialized in face processing^32^; (ii) M2, mainly localized in the left temporal cortex, can be supposed to be related to linguistic processing activated by the stimuli presented (participants were asked to focus on the “happy” vs “angry” verbal labels); (iii) M3 shows a bilateral temporal and parietal activity, possibly due to higher-order integration; (iv) M4 is mainly localized in the right frontal area, possibly representing the hemispheric specialization for the peculiar stimulus category.

### Microstates and emotional stimuli

**Figure 2:**
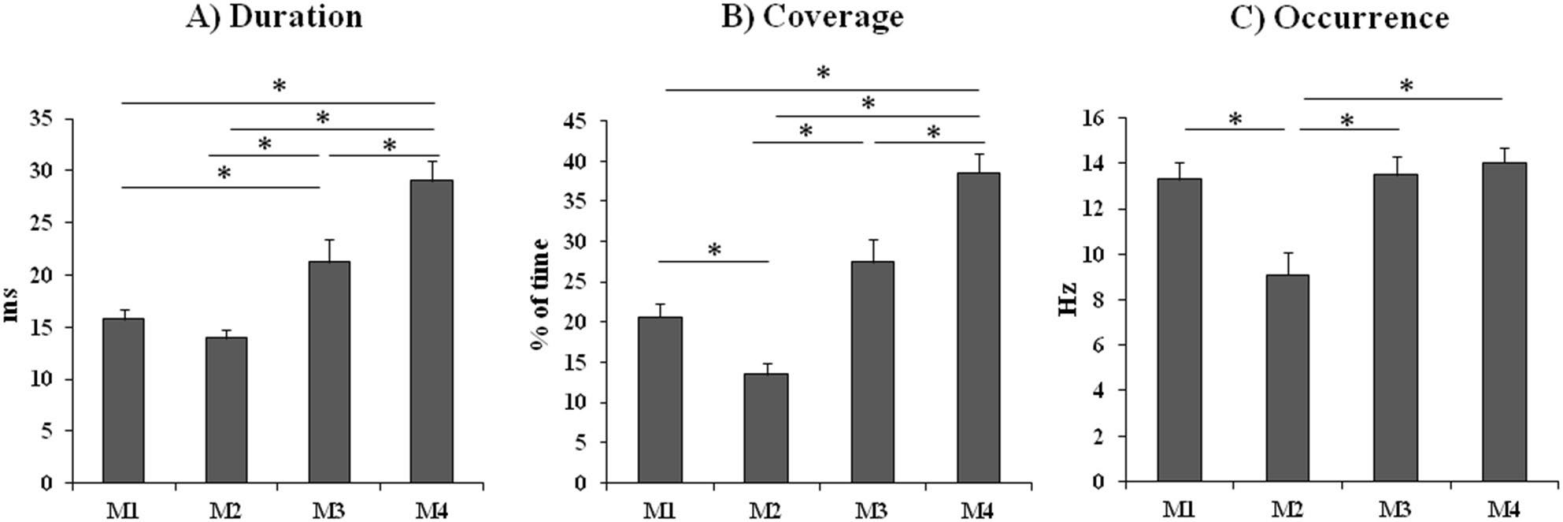
(**A**) Duration of M1, M2, M3, M4 (in milliseconds); (**B**) coverage of M1, M2, M3, M4 (in percentage of time); (**C**) occurrence of M1, M2, M3, M4 (in Hz). Bars represent standard errors and asterisks show significant comparisons.

In a first step, three ANOVAs were carried out, by using (i) duration, (ii) occurrence and (iii) coverage as dependent variables, and by using Microstate (M1, M2, M3, M4) as within-subject factor. The ANOVA on duration was significant (*F*_(3,45)_ = 20.26, *MSE* = 36.21, *p* < 0.001, *η*_*p*_^2^ = 0.57; see Fig. 2A) and post-hoc comparisons revealed that the duration of M4 was higher than all of the other microstates (*p* < 0.001 for all comparisons), and that the duration of M3 was higher than that of M1 (*p* = 0.013) and M2 (*p* = 0.002). The ANOVA on coverage was significant (*F*_(3,45)_ = 18.12, *MSE* = 100.01, *p* < 0.001, *η*_*p*_^2^ = 0.55; see Fig. 2B): post-hoc comparisons confirmed that also the coverage of M4 was higher than those of all of the other microstates (*p* < 0.005 for all comparisons), the coverage of M3 was higher than that of M2 (*p* < 0.001; a trend emerged also when compared with M1: *p* = 0.06), and that the coverage of M1 was higher than that of M2 (*p* = 0.048). Finally, also the ANOVA on occurrence was significant (*F*_(3,45)_ = 9.87, *MSE* = 8.32, *p* < 0.001, *η*_*p*_^2^ = 0.40; see Fig. 2C) and post-hoc comparisons showed that the occurrence of M2 was lower than those of all of the other microstates (*p* < 0.001 for all comparisons).

### Lateralized presentation of emotional faces

**Figure 3:**
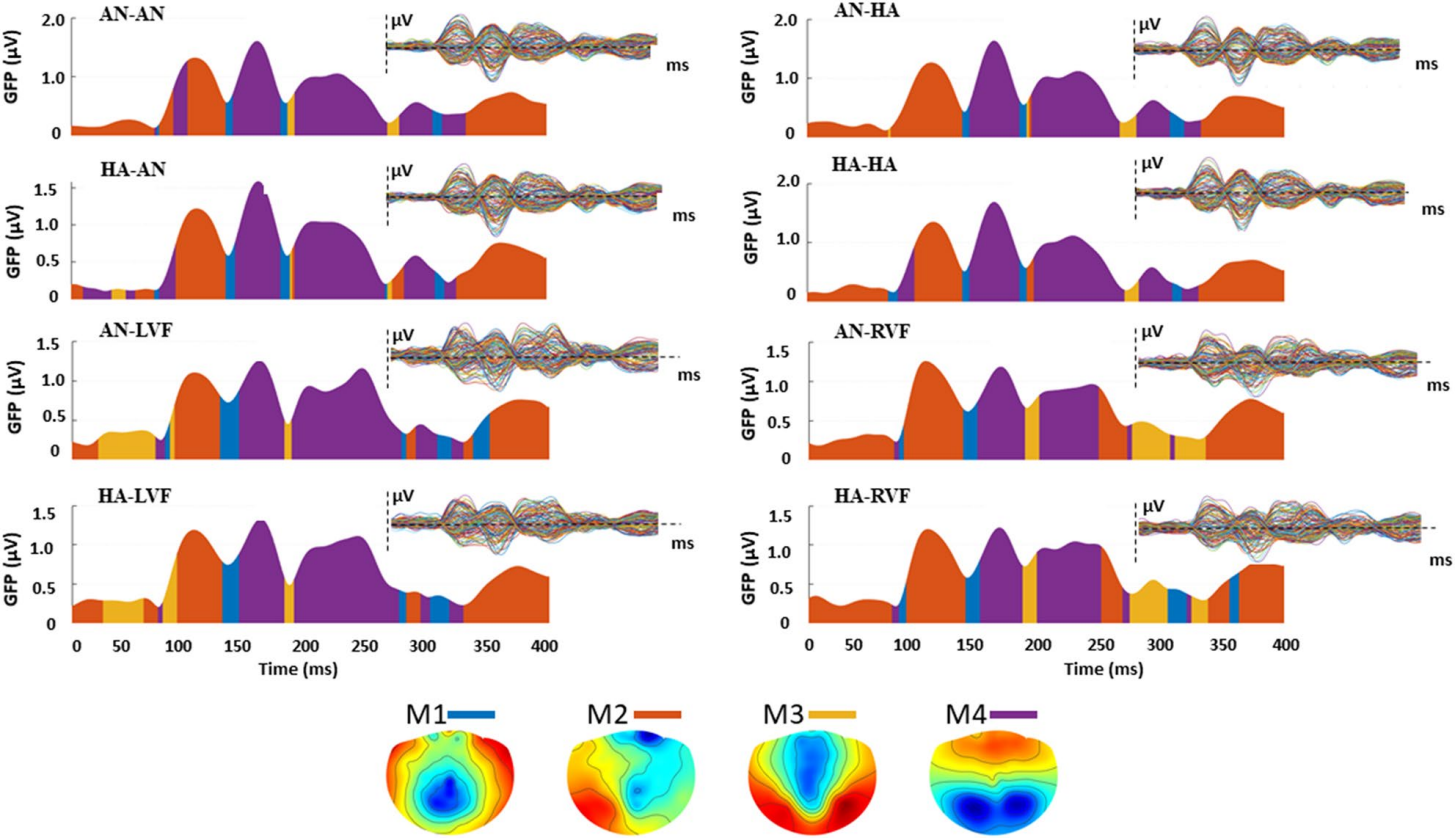
Graphical representation of temporal evolution of each microstate for each of the experimental conditions.

A second analysis focused on each single microstate, and was aimed at comparing each level for both the unilateral and the bilateral presentation, separately (see Fig. 3). An ANOVA with a single factor Condition (AN-AN, HA-HA, AN-HA, HA-AN) was carried out for the bilateral presentations, and a 2 × 2 ANOVA including Emotion (AN-HA) and Side (LVF, RVF) was carried out for the unilateral presentations (AN-LVF, AN-RVF, HA-LVF, HA-RVF). For each microstate no significant result emerged in the ANOVA on bilateral presentations. However, significant results emerged in the ANOVAs on the unilateral conditions.

#### Microstate 1

In the unilateral Emotion x Side ANOVA carried out on occurrence, the main effect Side was significant (*F*_(1,15)_ = 5.19, *MSE* = 3.49, *p* = 0.038, *η*_*p*_^2^ = 0.26). Post-hoc comparisons showed that the occurrence of M1 was higher when emotional faces were presented in the LVF (13.7 ± 0.68) than in the RVF (12.64 ± 0.64).

#### Microstate 2

For M2, the unilateral Emotion x Side ANOVA carried out on occurrence showed a significant main effect of Emotion (*F*_(1,15)_ = 7.63, *MSE* = 7.32, *p* = 0.015, *η*_*p*_^2^ = 0.34), with a higher occurrence of M2 for happy (11.02 ± 0.99) than for angry faces (9.15 ± 0.78).

#### Microstate 3

Concerning M3, the unilateral Emotion x Side ANOVA revealed a significant main effect of Side for both occurrence (*F*_(1,15)_ = 10.08, *MSE* = 12.84, *p* = 0.006, *η*_*p*_^2^ = 0.40), with higher values for stimuli presented in the RVF (14.7 ± 0.8) than in the LVF (11.85 ± 0.74), and for coverage (*F*_(1,15)_ = 15.60, *MSE* = 21.36, *p* = 0.001, *η*_*p*_^2^ = 0.51), with higher values for stimuli presented in the RVF (29.53 ± 2.43) than in the LVF (24.97 ± 2.32).

#### Microstate 4

In the unilateral Emotion x Side ANOVA of M4, the main effect of Side was significant both for duration (*F*_(1,15)_ = 4.87, *MSE* = 68.86, *p* = 0.043, *η*_*p*_^2^ = 0.25), with higher duration values for stimuli presented in the LVF (29.97 ± 2.12) than in the RVF (25.29 ± 1.87), and for coverage (*F*_(1,15)_ = 6.71, *MSE* = 14.2, *p* = 0.020, *η*_*p*_^2^ = 0.31), with higher values for faces presented in the LVF (36.93 ± 2.47) than in the RVF (34.49 ± 2.46).

### The effect of positive and negative valence on microstates

In a third analysis, microstates were investigated with respect to the emotional valence of the stimuli (positive: happy; negative: angry). Specifically, for each microstate (M1, M2, M3, M4) and for each dependent variable (duration, occurrence and coverage), two ANOVAs were carried out, one on negative valence and one on positive valence. In each ANOVA, five levels were considered: analyses on the negative valence included (i) an angry face in the left visual field (AN-LVF), (ii) an angry face in the right visual field (AN-RVF), (iii) two angry faces, one in each visual field (AN-AN), (iv) an angry face in the LVF during the simultaneous presentation of a happy face in the RVF (AN-HA), and (v) an angry face in the RVF, during the simultaneous presentation of a happy face in the LVF (HA-AN). Analyses on the positive valence included (i) a happy face in the LVF (HA-LVF), (ii) a happy face in the RVF (HA-RVF), (iii) two happy faces, one in each visual field (HA-HA), (iv) a happy face in the LVF, during the simultaneous presentation of an angry face in the RVF (HA-AN), and (v) a happy face in the RVF, during the simultaneous presentation of an angry face in the LVF (AN-HA).

No significant results emerged for negative valence, whereas significant difference emerged for positive valence for each dependent variable (duration, coverage and occurrence).

#### Duration

**Figure 4:**
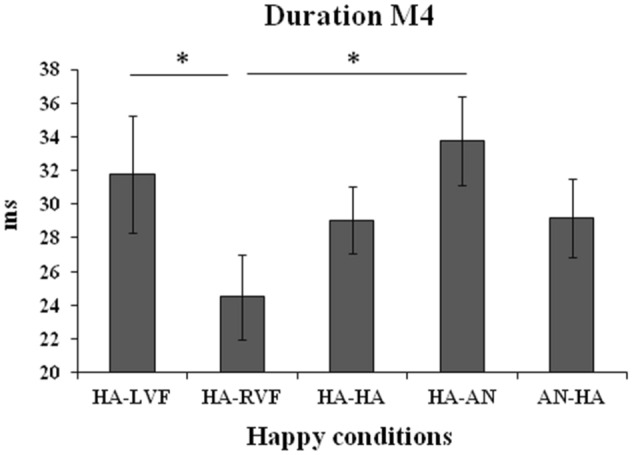
Duration of M4 (in milliseconds) on the five conditions in which the positive valence (happy face) is presented. Bars represent standard errors and asterisks show significant comparisons.

The ANOVA on duration was significant for M4 (*F*_(4,60)_ = 2.83, *MSE* = 69.13, *p* = 0.033, *η*_*p*_^2^ = 0.16; Fig. 4). Post-hoc comparisons showed that the duration of this microstate was lower for the unilateral condition HA-RVF with respect to both the HA-LVF (*p* = 0.025) and the bilateral HA-AN conditions (*p* = 0.005).

#### Coverage

**Figure 5:**
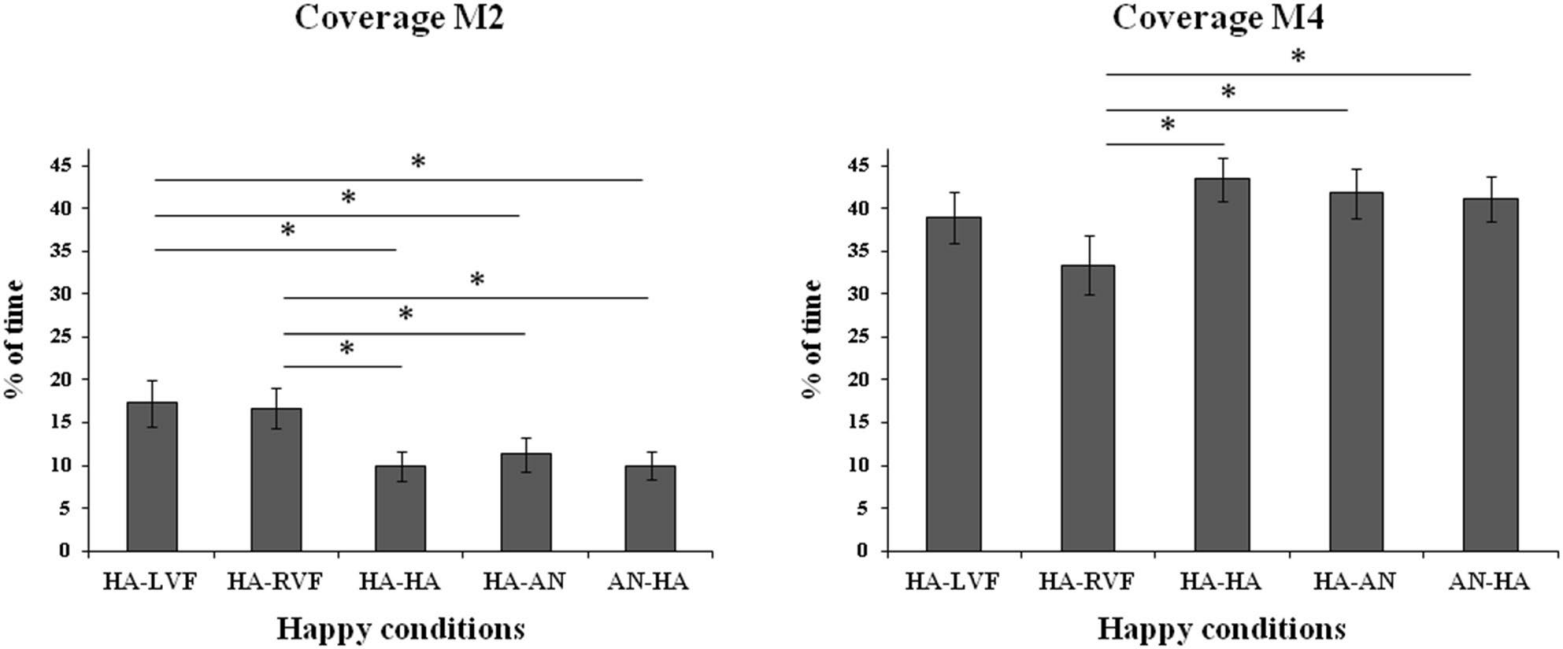
Coverage (in percentage of time) on the five conditions in which the positive valence (happy face) is presented, for M2 (left panel) and M4 (right panel). Bars represent standard errors and asterisks show significant comparisons.

The ANOVA on M2 was significant (*F*_(4,60)_ = 4.38, *MSE* = 49.24, *p* = 0.004, *η*_*p*_^2^ = 0.23; Fig. 5, left panel). Post-hoc comparisons showed that the coverage of M2 for both the left and right unilateral presentations of a happy face (HA-LVF and HA-RVF) was higher than all of the bilateral conditions (HA-HA, HA-AN, AN-HA, *p* < 0.035 for all comparisons).

The ANOVA on coverage was significant also for M4 (*F*_(4,60)_ = 3.69, *MSE* = 65.5, *p* = 0.009, *η*_*p*_^2^ = 0.20; Fig. 5, right panel) and post-hoc comparisons showed that the coverage of M4 was lower for the happy face presented unilaterally in the RVF (HA-RVF) with respect to all of the other conditions (HA-HA: *p* = 0.002, AN-HA: *p* = 0.012, HA-AN: *p* = 0.008, HA-LVF: almost significant with *p* = 0.055).

#### Occurrence

**Figure 6:**
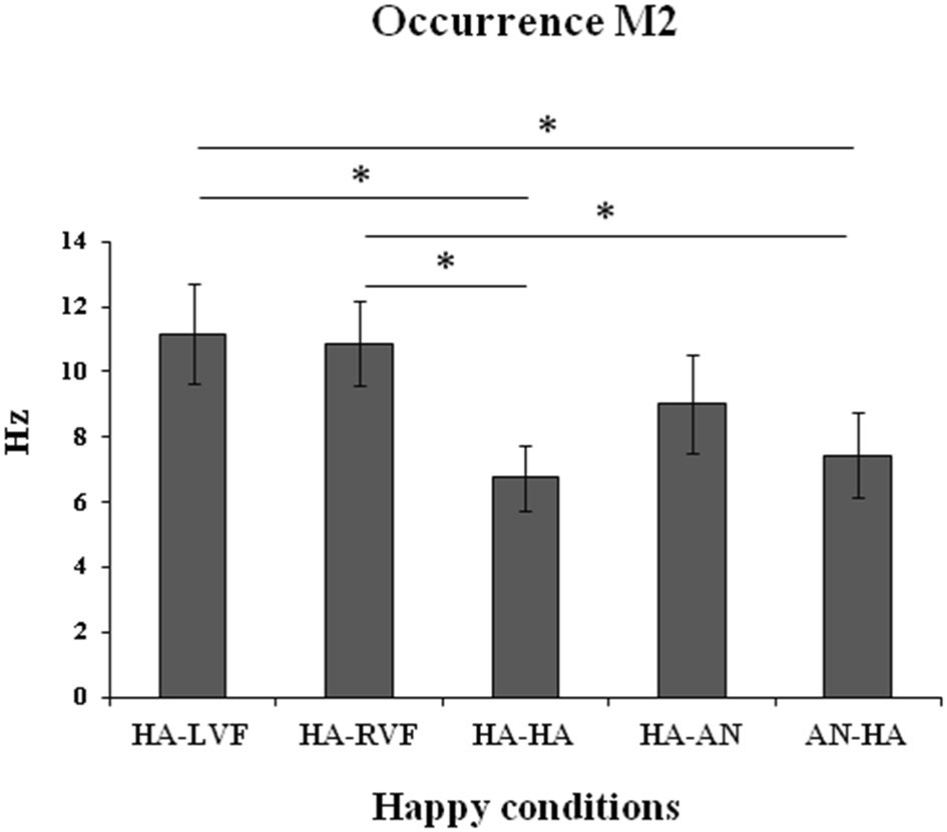
Occurrence of M2 (in Hz) on the five conditions in which the positive valence (happy face) is presented. Bars represent standard errors and asterisks show significant comparisons.

Finally, as regards occurrence, the ANOVA on M2 for the positive valence was significant (*F*_(4,60)_ = 4.31, *MSE* = 14.49, *p* = 0.004, *η*_*p*_^2^ = 0.22; Fig. 6). Post-hoc comparisons showed that the occurrence of M2 was higher for the two unilateral presentations (HA-LVF and HA-RVF) than for both the bilateral presentation of two happy faces (HA-HA, *ps* < 0.006) and the bilateral presentation of a happy face in the RVF and an angry face in the LVF (AN-HA, *ps* < 0.02). The remaining comparisons were not significant.

### The effect of handedness on microstates

**Figure 7:**
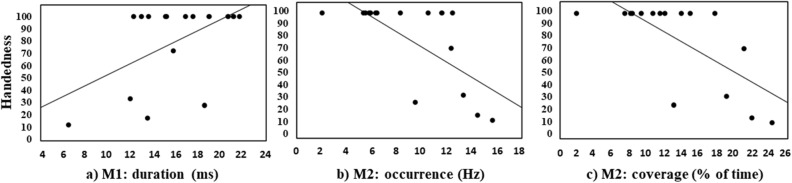
Correlations between handedness scores (higher values correspond to stronger rightward preference) and: (**a**) duration of M1 (left panel), (**b**) occurrence of M2 (central panel), and (**c**) coverage of M2 (right panel).

In a last exploratory analysis, correlations were computed between the handedness scores of the sample (with higher values corresponding to a stronger rightward preference) and each of the three dependent variables for each microstate (Fig. 7). Concerning M1, a positive correlation emerged when duration was considered (*r* = 0.51, *p* = 0.045), revealing that M1 showed a higher duration in the most extremely right-handed participants (Fig. 7a). Conversely, as concerning M2, a negative correlation emerged between handedness and both occurrence (*r* =  − 0.70, *p* = 0.003; Fig. 7b) and coverage (*r* =  − 0.71, *p* = 0.002; Fig. 7c), revealing that M2 showed a lower occurrence and coverage in the most extremely right-handed participants. No other significant correlations emerged.

## Discussion

The present study aimed at exploring the cerebral correlates of emotion processing by exploiting EEG microstate analysis. Starting from the contrasting literature about hemispheric asymmetries for positive/negative emotions, we aimed at disentangling the contribution of the two brain hemispheres during the presentation of one vs two emotional stimuli showing positive/negative emotions, by prying up on a method of signal analysis in which a priori hypotheses are not required, namely EEG microstates.

The first main result of the present study is the evidence of four microstates associated with the presentation of emotional faces: M1 and M2, mainly localized in the right and left temporal areas, respectively, M3 with a bilateral temporo-parietal distribution, and M4 with a right frontoparietal localization. These microstates were extracted in a period of 400 ms following the stimulus onset and can be considered in relation to the known fMRI resting state networks (RSN)^27^, at least partially. For instance, M1 could be partially overlapped to the RSN localized in the bilateral superior and middle temporal gyri, which has been suggested to be related to a phonological/auditory network (microstate A). This microstate was found also at rest, so that it has not to be considered as related to a linguistic task, rather as a RSN possibly associated with “basic” brain activity. Alternatively, it is well known that the temporal gyri are involved in facial emotion processing^33,34^, and this evidence, together with the temporal localization of the cerebral area specifically involved in face processing^32^, can justify the presence of the temporal microstates in the present task. Coming back to the RSN, M4 can be intended as overlapped to the circuit corresponding to the frontoparietal attentional network, mainly lateralized to the right hemisphere (microstate D). As concerning M2 and M3, however, they seem to be independent from the RSN previously described: in fact, if the literature on both fMRI and EEG networks at rest describes a bilateral occipital activity (microstate B) and a bilateral cingulate/right frontal network (microstate C)^27^, the present study does not show such patterns of activity. In our study, in fact, besides a temporal activity involving the right-lateralized Fusiform Face Area (M1)^32^ and the limbic structures, as well as the homologue area in the left hemisphere (M2), we found the involvement of a higher order temporo-parietal network (M3), and a specific frontoparietal network (M4). The present analyses on the metrics of the four microstates revealed that the duration and the coverage of M3 and M4 networks are higher than those recorded for M1 and M2 and that the occurrence of M2 is the lowest with respect to the other microstates. This pattern of results, as shown in Fig. 2, confirms that M3 and M4 are the specific networks related to the emotional detection required in this task, since they are more prominent with respect to the others, in terms of duration, coverage and occurrence.

Concerning the positive/negative valence of the stimuli, our results show that the only significant difference is that on the positive emotional expression (happy faces): in fact, no difference emerged in each microstate when the angry expression was presented, whereas they occurred for the happy expression. This result can be framed in the unresolved field of hemispheric asymmetries for positive and negative emotions. In fact, both main theories in this domain postulate the same right-hemispheric superiority for negative emotions, so that no difference were expected in this condition, but different patterns of activity are expected for happiness. Specifically, the presentation of a happy face should correspond to a stronger left-hemispheric activation according to the Valence Hypothesis (VH)^8,9^, and to a stronger right-hemispheric activation according to the Right Hemisphere Hypothesis (RHH)^6,7^. Besides having found higher coverage and occurrence for M2 during the unilateral than during the bilateral presentation of happy faces, the coverage of M3 was lower during the unilateral presentation of a happy face in the RVF (left hemisphere) than in all of the other unilateral and bilateral conditions. Moreover, the duration of M3 was lower during the unilateral presentation of a happy face in the RVF (left hemisphere) than during the presentation of a happy face in the LVF (right hemisphere) either alone (unilateral presentation) or together with an angry face in the opposite visual field (HA-AN). This pattern of results seems to suggest that M3 is less present when only a positive valence emotion is presented to the left hemisphere, in contrast with the VH. Further evidence in this regard comes from the statistical comparisons among the unilateral presentations: the main effect of Side (LVF vs RVF) was significant for both the duration and the coverage of M3, with higher values when emotional faces were presented in the LVF (right hemisphere) than in the RVF (left hemisphere), further confirming a main involvement of the right hemisphere in all emotions independently of their positive or negative valence and thus further supporting the RHH^6^. Similarly, the occurrence of M1 was higher for stimuli presented in the LVF than in the RVF. Nevertheless, the right-lateralized frontoparietal network constituting M4 showed higher occurrence and coverage for stimuli unilaterally presented in the RVF than in the LVF. The fact that this network is anyway lateralized to the right hemisphere, together with the evidence that a microstate’s activity could correspond to a task-negative network, in which activity decreases—rather than increases—during the performance of a cognitive task^35–37^, allows us speculate that such a reduction of occurrence and coverage of M4 for faces presented in LVF can represent a specific task-related microstate, specifically showing the main involvement of the right hemisphere in emotion processing, independently of the valence. An alternative explanation is that M4 corresponds to the frontoparietal attentional network^17,27,36^ and that the presentation of an emotional face in the RVF (left hemisphere) leads to an increased attentional effort with respect to the direct presentation of the same stimulus to the right hemisphere, further supporting the main role of the right half of the brain in emotion processing^6,12,30^.

Finally, starting from previous results revealing that participants with a strongest right hand preference expressed faster and more positive emotional judgments for happy faces presented in the RVF (left hemisphere) than in the LVF^22^, here we also explored the possible correlation between handedness and the metrics of the four microstates, separately. This exploratory analysis showed a positive correlation between the rightward motor preference and the duration of M1, as well as a negative correlation between the rightward preference and both occurrence and coverage of M2. These results, together with the previous ones, suggest a possible role of handedness on the cerebral processing of emotions (see also^38–40^). However, the fact that the sample was composed only of right-handed participants, with non-normally distributed data, led us to consider this result as suggestive evidence in this regard, but further studies are needed in order to shed light on this possible link.

To conclude, our results revealed four microstates associated with the task of passively observing facial expressions: M1 and M2 mainly involving the limbic and temporal areas, M3 including temporo-parietal sites and M4 with a frontoparietal distribution. The specific results found for duration, occurrence and coverage associated with each microstate led us to conclude that (i) microstates are a tool useful not only for analyzing resting states, but also task-related data, (ii) M3 and M4 seem to be the microstates more responsive to the emotional stimuli, with higher duration, occurrence and coverage than the other microstates, (iii) their parameters differ among conditions and the results revealed that in particular M4 is dependent upon the positive emotion presented in a visual hemifield. This peculiar frame confirms that for negative emotions no difference emerges in the brain activity according to the lateralized presentation of the stimuli, whereas positive emotions are related to a different pattern of cerebral networks, in accordance with the specific hemisphere directly stimulated. In this specific study, by using this innovative approach, we found further support for the main role of the right hemisphere in emotion processing, corroborating previous evidence collected by means of standard ERP analysis and EEG connectivity^15,30^. It has to be highlighted, however, that further studies are needed to corroborate our conclusion: due to the innovative statistical method used here and to the amount of comparisons carried out, indeed, caution is required in the interpretation of the results, also due to the low sample size—even if it has been shown that a high number of repetitions for each condition, as used in the present study, can compensate at least in part for this problem^41^.

As regard previous results with EEG microstates, a difference between neutral and emotional words has been already described^28,29^, but to our knowledge this is the first study in which EEG microstates have been extracted during a divided visual field paradigm with emotional faces, and our results suggest this method is a valid tool to collect new evidence of brain activity during a specific task. Concerning the dispute between the RHH and the VH, the present pattern of results—albeit complex—mainly support the RHH, adding new evidence to this contrasting field. Further studies are needed to better reveal the usefulness of this approach in shedding light on the cerebral basis of different cognitive domains, and the results found here can be intended as a first step in this direction. The fact that the present results support the main involvement of the right hemisphere in emotion processing, without the need of an a priori hypothesis, makes EEG microstates a potential instrument to investigate hemispheric asymmetries also in clinical conditions. Indeed, altered hemispheric activity has been described in different clinical conditions such as depression^42–44^, anxiety^45,46^, autism^47,48^ and schizophrenia^49–52^ with respect to controls, and we suggest that, starting from this frame, EEG microstates can become a useful tool for further exploring the hemispheric imbalance, both at rest and during a task, in these clinical populations. Finally, in the present study a positive (happy) and a negative (angry) expression were used, as in previous EEG studies in the same domain^15,22,30^, but a wider range of emotions should be considered in future studies. In fact, besides the two main hypotheses on hemispheric asymmetries for emotions considered here (RHH and VH), a different model of hemispheric involvement was also suggested, linked to the approach/avoidance reaction elicited by an emotional stimulus which would lead to a stronger left/right-hemispheric frontal activity^53^. In this view, albeit anger and happiness are different in valence, they are both approach-related, so that different patterns of results could be found comparing these expressions with avoidance-related/inhibition expressions (i.e., fear or sadness). Nevertheless, the present evidence of a strong right-hemispheric activity for both anger and happiness, together with previous evidence collected with the same paradigm^15,22^, did not support the left-hemispheric superiority for approach-related emotions, but direct comparisons among all the basic emotional expressions would add important evidence in the complex literature about the cerebral bases of emotion processing.

## Material and methods

### Procedure

A sample of 16 healthy volunteers (mean age: 27.25 ± 0.93 years; nine females) participated in the experiment (same sample as in Prete et al.^22^, in which a sample size calculation was carried out by means of G-Power software). All participants were right-handers, as measured by the Edinburgh Handedness Inventory (Italian version^54^) with a mean handedness score of 78.85 (± 8.64), in a range from − 100 (complete left preference) to 100 (complete right preference). Participants were presented with photographs in frontal view of 15 female and 15 male faces in happy and angry poses. All stimuli were equated for size and luminance and were presented in gray scale. A total set of 960 trials was divided in half trials in which one emotional face was presented (unilateral presentations) and the other half in which two emotional faces were presented together (bilateral presentations). In unilateral presentations, either an angry (AN) or a happy (HA) face was presented in either the Left Visual Field (LVF) or in the Right Visual Field (RVF), and a black and white checkerboard (with the same size and luminance as facial stimuli) was presented in the opposite visual field, for a total set of four conditions: AN-LVF, AN-RVF, HA-LVF, HA-RVF. In the bilateral presentations, two faces were presented together, one in each visual field. In these conditions the two stimuli could show either the same emotion or the two different emotions, for a total set of four conditions with different combinations of emotions in the LVF-RVF, as follows: HA-HA, AN-AN, HA-AN, AN-HA (see also^15^).

**Figure 8:**
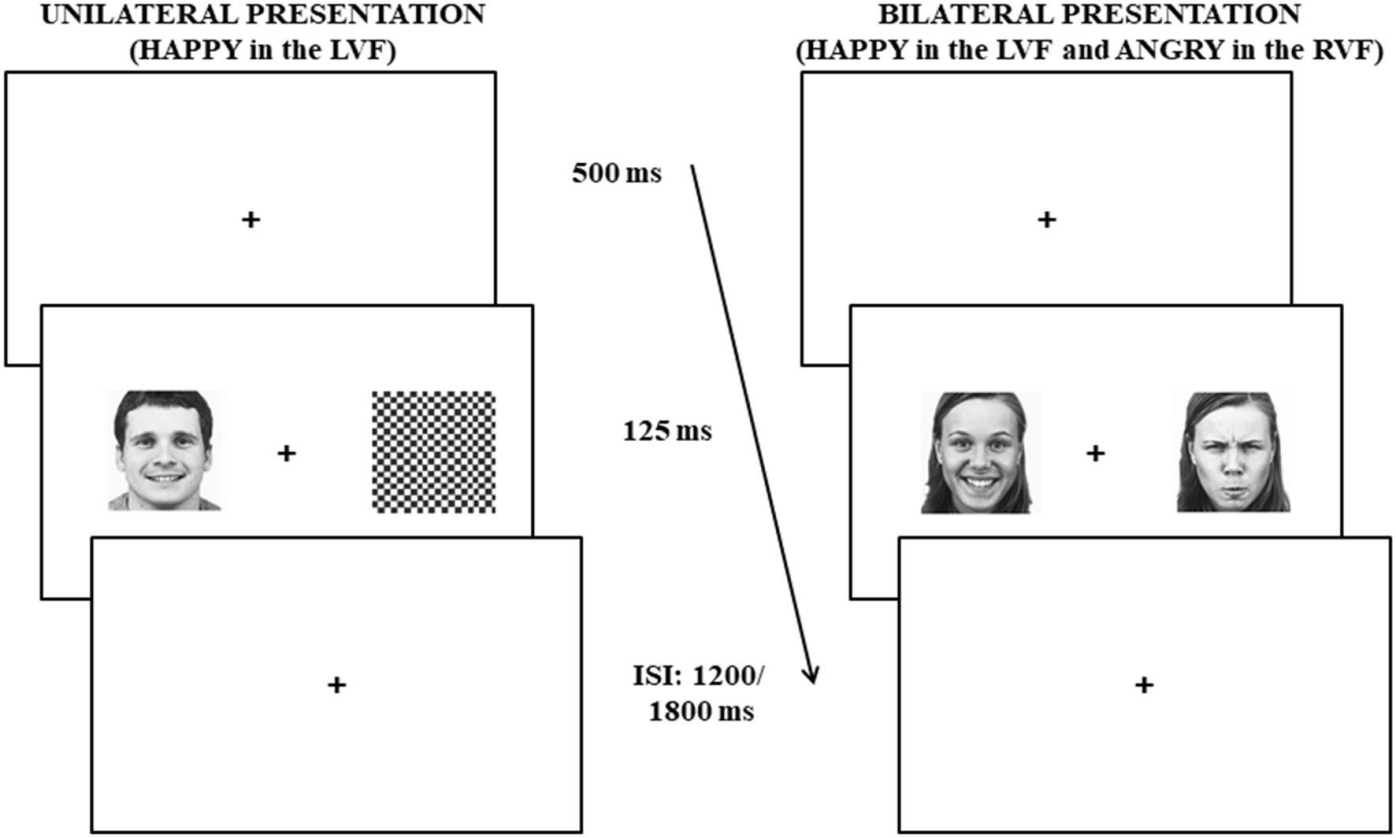
Schematic representation of the timeframe of a trial. *Left panel* example of unilateral presentation; *right panel* example of bilateral presentation.

In each trial, after the presentation of a black fixation cross in the center of the screen for 500 ms, a stimulus was presented for 125 ms (either one emotional face and the checkerboard, or two emotional faces; see Fig. 8), with its center placed at 9.19° of visual angle to the left or to the right of the center of the screen. During the following inter-stimulus interval, which was randomized between 1200 and 1800 ms, a fixation cross was presented.

The eight conditions (four unilateral and four bilateral) were passively repeated 120 times each. Moreover, 128 further trials were used to collect behavioural responses: participants were instructed to judge the emotional expression of the stimulus by using a 5-points Likert scale (from 1 = very angry, to 5 = very happy). These trials requiring a behavioural response were included in the original protocol^22^, but they are excluded from the present analysis. The order of the trials was randomized within and across participants and the whole task lasted about 40 min.

During the entire procedure, EEG was recorded by means of a 128-electrode net (Electrical Geodesic, Version 1.1), placed according to an augmented 10–20 system, with the skin–electrode impedance kept below 50 kΩ^55^. Informed consent was obtained from all participants prior to testing, the procedure was carried out in accordance with the principles of the Declaration of Helsinki, and was approved by the Ethics Committee for Biomedical Research of the University “G. d’Annunzio” of Chieti and Pescara.

### Microstates computation and statistical analysis

Microstate analysis can be conceived as a method to uncover periods of topographical stability (40–120 ms) from the EEG signals. Indeed, applying a space reduction using a cluster algorithm allows to extract a certain number of fixed topographies (global templates), called microstates. By fitting such topographic maps to the EEG time course, it is possible to obtain a discrete sequence of microstates. Microstate analysis is suitable for both ongoing and ERP experiments. In the latter case, microstates backfitting is based on the ERP of each participant^56^.

In order to find global templates across conditions, in this study, the clustering algorithm was applied concatenating data from all conditions. We considered a period of 400 ms following the target onset for the ERP analysis, and for each subject’s ERP from each condition, all maps for each time course point^57^ were submitted to a modified version of k-means clustering algorithm considering the polarity of the maps^58^. The k-means algorithm was repeated varying k from 1 to 12. The optimal number of k was chosen by estimating, for each subject and for each condition repetition, the Krzanowski-Lai (KL) criterion and by choosing the number of clusters corresponding to the second KL maximum value^59^.

Next, we grouped all subject-wise maps obtained in the previous step and performed a second k-means clustering, varying again k from 1 to 12^60^. For each participant, the maps identified through such procedure (global templates) were fitted back to the ERPs of each condition (AN-LVF, AN-RVF, HA-LVF, HA-RVF, AN-AN, HA-HA, AN-HA, HA-AN) assigning each time frame to templates that best fit in terms of spatial correlation, obtaining a microstates sequence. The back-fitting procedure provided for each ERP a temporal sequence of microstates, that were used to calculate metrics depending on the conditions:

1. mean microstate duration: the average time covered by a single microstate class;
2. mean occurrence per second: mean number of distinct microstates of a given class;
3. mean percentage of covered analysis time: percentage of time covered by a single microstate class.

Moreover, to determine the underlying cortical sources, the eLORETA^61^ approach was applied to each microstate’s topography (M1, M2, M3, M4). The volume conductor model was given by a boundary element method (BEM^62^) of a template brain (http://www.bic.mni.mcgill.ca/ServicesAtlases/Colin27) and the source space was modeled by a Cartesian 3D grid bounded by the template anatomy with 5113 voxels. Visualization of cerebral sources was done by means of the connectome workbench (https://www.humanconnectome.org/software/connectome-workbench).

At a statistical level, data were analyzed by means of analyses of variance (ANOVAs). Statistical significance threshold was set at *p* = 0.05 and, when needed, post-hoc comparisons were computed by means of Duncan test^63^ (as in the previous analyses on the same data^15,22^).

## Data Availability

The datasets generated during and/or analyzed during the current study are available online at https://drive.google.com/file/d/1vKot7lmYfof-KFlf1aDKDZyNcQ3ixqdq/view?usp=sharing.
